# Biodegradation of Pollutants in Waste Water from Pharmaceutical, Textile and Local Dye Effluent in Lagos, Nigeria

**DOI:** 10.5696/2156-9614-6.12.34

**Published:** 2016-12-19

**Authors:** Idowu A. Aneyo, Funmilayo V. Doherty, Olumide A. Adebesin, Mariam O. Hammed

**Affiliations:** 1 Environmental Biology Unit, Department of Biological Science, Yaba College of Technology, Yaba, Lagos, Nigeria; 2 Cell Biology, University of Lagos, Akoka, Lagos, Nigeria

**Keywords:** biodegradation, pollutant, heavy metal, wastewater

## Abstract

**Background.:**

Discharged effluents from industry have been responsible for the deterioration of the aquatic environment in many parts of the world, especially in developing countries. Increasing industrialization and urbanization have resulted in the discharge of large amounts of waste into the environment, resulting in high pollution loads. Utilization of microbes such as fungi and bacteria have been used for pollution degradation.

**Objectives.:**

The aim of this research was to utilize microbial agents such as fungi and bacteria to reduce pollutant loads such as heavy metals in effluent samples.

**Methods.:**

Three types of effluent (pharmaceutical, textile effluent, and dye) were obtained from Surulere in Lagos Metropolitan Area, Nigeria. Heavy metals analysis was carried out using a flame atomic adsorption spectrophotometer according to standard methods. Samples were cultured for microbes and identified. Bacteria samples were inoculated on nutrient agar and incubated at 37°C for 24 hours. Fungi counts were carried out using potato dextrose agar and incubated at 28°C for 3–5 days. The isolated organisms were identified based on their morphological and biochemical characteristics. Then 100 mL of the effluents was dispensed into 250 mL flasks, and the pH of the medium was adjusted to 7.2 by the addition of either sodium hydroxide or hydrogen chloride and autoclaved at 121°C for 15 minutes. The autoclaved flask was inoculated with 1 mL of bacteria and fungi for 21 days and pH was recorded properly every 48 hours.

**Results.:**

The results of the physicochemical parameters indicated that conductivity, total suspended solids, total dissolved solids, turbidity, chemical oxygen demand and biochemical oxygen demand for all the three industrial effluents were higher than the World Health Organization (WHO) permissible limits. Heavy metal analysis results show that the effluents had high values for cadmium, above the WHO limit of 0.003 mg/L. Concentrations of zinc ranged from 0.136–1.690 mg/L, and nickel ranged between 0.004–0.037mg/L for the three effluents, within the WHO limit. The identified bacteria were Bacillus subtilis, Klebsiella pneumonia, Salmonella typhi and Bacillus cereus and isolated fungi were Aspergillus flavus and Penicillium chrysogenum. All the physicochemical parameters and heavy metal concentrations were reduced after the biodegradation study in the effluents.

**Conclusions.:**

The responses observed in the various microbes indicated that the use of microbes for the reduction of environmental pollutants has an advantage over the use of other methods because it is environmentally friendly, low cost, and no new chemicals are introduced into the environment. This method should be encouraged for pollution reduction to bring about ecosystem sustainability advocated for Ghana.

## Introduction

Microorganisms have been utilized in environmental remediation for decades. Bioremediation is defined as the use of biological agents such as bacteria, fungi, or green plants (phytoremediation) to remove or neutralize hazardous substances in polluted soil or water. 1,2 Increasing human population has led to an increase in industrial activities. One of the main sources of pollution worldwide is the textile industry and its dye-containing wastewaters. About 25% of textile dyes are lost during the dyeing process, and 2–20% are discharged as aqueous effluents in different environmental components. The discharge of dye-containing effluents into the water environment is undesirable because of its colour, direct release and its breakdown products are toxic, carcinogenic or mutagenic to life forms due to carcinogens such as benzidine, naphthalene and other aromatic compounds.^3^ The textile industry generates a high volume of waste water with the potential for water pollution. Among the many chemicals in textile waste water, dyes are major pollutants.^4^

**Abbreviations**
*BOD*: Biochemical oxygen demand
*COD*: Chemical oxygen demand
*WHO*: World Health Organization

Environmental problems such as appearance of colour in discharges from various industries, combined with the increasing cost of water for the industrial sector, have made the treatment and reuse of effluent increasingly attractive to the industry.^5^

The textile industry is one of the oldest industries in India with over 1000 factories. Due to the volume and composition of its effluent, textile wastewater is considered to be the most polluting among all of the industrial sectors.^6^,^7^ Wastewater from a typical textile plant is characterized by high values of biochemical oxygen demand (BOD), chemical oxygen demand (COD), colour and pH.^8^,^9^ It is a complex and highly variable mixture of many polluting substances ranging from inorganic compounds and elements to polymers and organic products.^10^ The incomplete use of dye and washing operations result in textile wastewater retaining a considerable amount of dye.^11^

Pharmaceutical wastewater is a complex mixture of different organic and inorganic compounds, including residues of active pharmaceutical substances, solvents, and toxic and bio-recalcitrant chemicals that inhibit microbial activity of the activated sludge process and present a great challenge for the proper treatment and downstream processing of wastewater.^12^ In the pharmaceutical industry, wastewater is mainly generated through equipment washing activities. Although the wastewater discharged is small in volume, it is highly polluted due to the presence of substantial amounts of organic pollutants. Levels of wastewater pollution vary from industry to industry, depending on the type of process and the size of the industry.^13^ Typically, pharmaceutical wastewater is characterized by a high COD concentration and some pharmaceutical wastewaters have COD levels reaching as high as 80.000 mg/L. Pharmaceutical companies are one of the major contributors of hazardous and toxic effluents. Ireland alone generates about 43 tons BOD in its pharmaceutical industry.^14^ The recycling of treated wastewater has been recommended due to the high levels of contamination stemming from dyeing and finishing processes (i.e. dyes and their breakdown products, pigments, dye, intermediate, auxiliary chemicals and heavy metals).^15–18^

The aim of this research was to utilize microbial agents such as fungi and bacteria to reduce pollutant loads such as zinc, cadmium, and nickel in effluent samples.

## Methods

### Sampling Location

Effluents were collected in Surulere in Lagos Metropolitan Area, Nigeria from different industries from their main sites. Surulere is a commercial area where many manufacturing industries are located. The coordinates of the sample locations are presented in Table 1. Lagos Metropolitan Area is a megacity and contains 70% of the industries of Nigeria.

**Table 1:** Coordinates of the Sample Locations

**Industry**	**GPS Readings**
Pharmaceutical	Longitude N06° 31.22, Latitude E003° 14.15
Textile	Longitude N06°29.135, Latitude E003° 21.232
Dye	Longitude N06°29.396, Latitude E003° 19.960

### Sample Collection

Effluent samples were collected at the discharge pipe at about 7:30 am with three replicates (A1, A2, A3, etc.) from pharmaceutical, textile and dye industries. The samples were collected in sterile sample bottles and transported immediately to the laboratory and stored at around 4°C. Then, 250 mL samples were collected and put in sterile reagent bottles (500 mL capacity). The samples were subjected to immediate physicochemical analysis on site. These samples served as the source for the isolation of micro-organisms.

### Physicochemical and Heavy Metal Analysis of Effluents

All samples were analyzed for heavy metals (zinc, cadmium, and nickel) and physicochemical parameters according to internationally accepted procedures and standard methods.^19^,^20^ The analyzed parameters included temperature, chemical oxygen demand, dissolved oxygen, biochemical oxygen demand, turbidity, odour, colour, total suspended solids, pH, conductivity, and total dissolved solids. In addition, pH, temperature, and dissolved oxygen were determined on site using appropriate meters (pH meter hanna HI9813, TDS-3 HM digital for temperature, and DO analyser model JPSJ-605). The concentrations of heavy metals were determined using an atomic absorption spectrophotometer.

### Microbial Analysis

#### Total Bacterial Count

The collected samples were analysed for the presence of microorganisms. First, 1 mL of each effluent sample was transferred into 9 mL of sterile saline solution in a test tube and shaken vigorously. The solution was serially diluted and 10^−3^ dilution was taken and plated using the pour plate technique on Petri dishes. The bacteria were inoculated on nutrient agar and incubated at 37°C for 24 hours. This was carried out using procedures which have been previously reported.^21^

#### Total Fungal Count

Fungal counts were conducted using potato dextrose agar with 10% tartaric acid using the spread plate method. This was carried out according to previously reported methods.^22^ Microbial count of the effluents samples were reported as colony forming units per gram (cfu/g).

#### Characterisation and Identification of Organisms

The identification of bacteria was based on biochemical characterizations including citrase, urease, catalase, indole, raffinose, xylose, galactose, starch hydrolyses, and oxidase reaction. The macroscopic colonial appearances of fungal growth in plates were observed and recorded. The macroscopic examinations were based on colony texture, size, pigmentation, time of growth, color on the reverse side of the plate and colony margin.^23^ A drop of lactophenol cotton blue was placed on a grease free, scratch-free glass slide.^24^ A small portion of the fungal growth was picked with a wire loop and teased out using a mounting needle. The preparation was covered with a cover slip.^25^ The slide was observed under 10× and 40× objective lenses. Observed characteristics were recorded and compared with the established identification keys as previously described.^26^

### Biodegradation of Effluents

#### Bacteria

Mineral salt medium prepared with the following composition was used for the studies: disodium phosphate (1.065 g) ammonium chloride (0.25 g), magnesium sulfate heptahydrate (0.10 g), monopotassium phosphate (0.65 g), and added to 500 mL of the effluents. Then, 100 mL of the effluents was dispensed into 250 mL flasks, the pH of the medium was adjusted to 7.2 by addition of either sodium hydroxide or hydrogen chloride, and autoclaved at 121°C for 15 minutes. The autoclaved flask was inoculated with 1 mL of bacteria inoculum of the microorganism and the flask was incubated for 21 days. The pH was recorded every 48 hours.

#### Fungi

Mineral salt medium prepared with the following composition was used for the studies: disodium phosphate (1.065 g), ammonium chloride (0.25 g), magnesium sulfate heptahydrate (0.10 g), monopotassium phosphate (0.65 g), and added to 500 mL of the effluents. Then, 100 mL of the effluents was dispensed into 250 mL flasks, the pH of the medium was adjusted to 5.6 by addition of either sodium hydroxide or hydrogen chloride, and chloramphenicol was added and autoclaved at 121°C for 15 minutes. The fungi plate was emulsified with 10 mL of sterilized distilled water, and then 1 mL of the fungal inoculum was inoculated into each autoclaved flask. The flasks were kept in the mechanical shaker and incubated at room temperature for 21 days. The pH was recorded at 3-day intervals.

## Results

### Physicochemical Parameters of Effluents

The results of the physicochemical parameters show that conductivity, total suspended solids (TSS), total dissolved solids (TDS), turbidity, COD and BOD for all the three industrial effluents were higher than the World Health Organization (WHO) permissible limits for water quality.^27^ The effluent from the local dye industry had the highest values for pH (12.02), conductivity (24500 m scm^−1^), DO (10 mg/L), TSS (7100 mg/L), TDS (12.500 mg/L), COD (290 mg/L), and BOD (150 mg/L), compared to effluents from the other two industries.

The pH of pharmaceutical and textile effluents were within the WHO permissible limits.^27^ Physical observation revealed the colour of pharmaceutical effluent, textile effluent and local dye effluent to be yellowish, black and reddish-brown, respectively. Odour was observed to be choky for pharmaceutical effluent and pungent smell was observed for textile and local dye effluents. Heavy metal analysis results show that the effluents had high values for cadmium, above the WHO limit of 0.003 mg/L. The concentration of zinc ranged from 0.136–1.690 mg/L, and the concentration of nickel ranged from 0.004–0.037 mg/L for the three effluents, all within the WHO limit.

**Table 2:** Characterization of the Three Wastewater Samples Compared to Permissible Limits

***Parameters***	***Pharmaceutical effluent***	***Textile effluent***	***Local dye effluent***	***WHO^27^,^28^ or Standard Organisation of Nigeria (SON)^29^ Federal Environmental Protection Agency - Nigeria (FEPA)^29^ permissable limits***
***pH***	6.00	6.89	12.02	6.5–8.5^27^,^28^
***Temperature (^°^C)***	27.31	26.72	26.4	<35^27^,^28^
***Conductivity (μScm^−1^)***	12900	6680	24500	200^27^,^28^
***DO (mg/l)***	8.17	5.03	10	8-5^27^,^28^
***Turbidity (Formazin Turbidity Unit)***	800	795	3550	—
***TSS (mg/L)***	1000	1400	7100	>10^27^,^28^
***TDS (mg/L)***	8290	4250	12500	500^27^,^28^
***COD (mg/L)***	125	109	290	10^27^,^28^
***BOD (mg/L)***	60	51	150	2–5^27^,^28^
***Zinc (mg/L)***	0.139	0.136	1.690	3.0^29^
***Cadmium (mg/L)***	0.337	0.183	0.030	0.003^27^,^28^
***Nickel (mg/L)***	0.004	0.037	0.012	0.02^27^,^28^
***Colour***	Yellowish	Black	Reddish brown	—
***Odour***	Choky	Pungent	Pungent	—

Abbreviations: DO, Dissolved oxygen; TSS, Total suspended solids; TDS, Total dissolved solids, a-World Health Organisation standard, b-Federal Environmental Protection Agency.

### Biochemical Identification of Bacterial Isolates

The results of the biochemical test are presented in Table 3. The identified bacteria included Bacillus subtilis, Klebsiella pneumonia, Salmonella typhi and Bacillus cereus. The isolates were Gram-positive rod for all samples from pharmaceutical and local dye effluent, and Gram positive and Gram negative rod for samples from textile effluent. Isolates were all negative for urease for samples from pharmaceutical effluent and negative for hydrogen sulfide gas production and indole for all samples from the three effluents.

**Table 3:** Biochemical Test of Bacterial Isolates

***Serial no***	***Gram reaction***	***Slant***	***Butt***	***H2S***	***Gas***	***Motility***	***Indole***	***Urease***	***Citrase***	***Oxidase***	***Catalase***	***Starch***	***Glucose***	***Galactose***	***5DI QRVH***	***Xylose***	***Organisms***
A1	GPR			−	−	+	−	−	+	+	−	+	+	−	+	−	Bacillus subtilis
A2	GPR			−	−	+	−	−	+	+	−	+	+	−	+	−	Bacillus subtilis
A3	GPR			−	−	+	−	−	+	+	−	+	+	−	+	−	Bacillus subtilis
B1	GNR	+	+	−	+	−	−	+	+	−	−						Kleb pneumoniae
B2	GNR	−	+	−	+	+	−	−	+	−	−						Salmonella typhi
B3	GPR			−	−	+	−	+	−	+	−	−	+	−	−	−	Bacillus cereus
C1	GPR			−	−	+	−	+	−	+	−	−	+	−	−	−	Bacillus subtilis
C2	GPR			−	−	+	−	−	+	−	−	+	+	−	+	−	Bacillus subtilis
C3	GPR			−	−	+	−	+	−	+	−	−	+	−	−	−	Bacillus cereus

Abbreviations: GPR, Gram positive rod; GNR, Gram negative rod; A, Pharmaceutical effluent; B, Textile effluent; C, Dye effluent.

### Morphological Characteristics and Identities of Isolated Fungi Associated with Biodegradation

The isolated fungi were Aspergillus flavus and Penicillium chrysogenum. The colony morphology and microscopic characterization are presented in Table 4.

**Table 4:** Morphological Characteristic and Identities of Isolated Fungi

***Serial No.***	***Morphological characteristics***	***Identified fungi***
***A1***	Pale brown with a smooth oval shaped conidia	**Aspergillus flavus**
***A2***	Creamish yellow with short smooth stipes and spherical conidia	Penicillium chrysogenum
***B2***	Pale brown with a smooth oval shaped conidia	**Aspergillus flavus**
***B2***	Creamish yellow with short smooth stipes and spherical conidia	Penicillium chrysogenum
***C1***	Creamish yellow with short smooth stipes and spherical conidia	Penicillium chrysogenum
***C2***	Creamish yellow with short smooth stipes and spherical conidia	Penicillium chrysogenum

Abbreviations: A, pharmaceutical effluent; B, textile effluent; C, dye effluent.

### Biodegradation

#### Bacteria

The microorganisms used for biodegradation include Bacillus subtilis for sample A (pharmaceutical effluent), Salmonella typhi for sample B (textile effluent) and Bacillus cereus for sample C (Dye effluent). There was a reduction in pH of the three effluents after 21 days. The pH of the pharmaceutical effluent reduced from 7.20 – 6.70, pH of the textile effluent reduced from 7.20-6.94, and pH of the dye effluent reduced from 7.20 – 7.00 after 21 days.

Results of the physicochemical analysis and heavy metal concentrations of the three effluents before and after biodegradation are presented in Table 5. All the physicochemical parameters and heavy metal concentrations were reduced after the biodegradation study (*Table 5*). Reductions of 18.41%, 9.0%, and 32.00% were recorded for conductivity in the pharmaceutical, textile and dye effluent, respectively. There was a 55.94%, 32% and 62% reduction in dissolved oxygen in the pharmaceutical, textile and dye effluent, respectively. A reduction of 13.75%, 11%, and 29% was recorded for turbidity in pharmaceutical, textile and dye effluent respectively. There was a reduction of 38%, 82.5% and 26% for total suspended solids in the pharmaceutical, textile and dye effluent, respectively. Total dissolved solids value showed a 62%, 28% and 33% reduction in pharmaceutical, textile, and dye effluent, respectively. A reduction of 51%, 43.05%, and 78% was recorded for COD in the pharmaceutical, textile and dye effluent, respectively. A reduction of 46%, 35%, and 77% was recorded for BOD in the pharmaceutical, textile and dye effluent, respectively. There was a reduction of 63%, 48%, 53% for cadmium, 50%, 56%, 58% for nickel, and 41%, 20%, 36% for zinc in pharmaceutical, textile and dye effluents, respectively.

**Table 5:** Physicochemical Analysis and Heavy Metal Concentrations of the Three Effluents Before and After Biodegradation

***Parameters***	***Pharmaceutical effluent***		***Textile effluent***		***Dye effluent***	
	Before	After	Before	After	Before	After
***pH***	6.0	6.67	6.89	6.53	12.0	6.68
***Temperature (°C)***	27.31	25.0	26.72	25.50	26.40	25.40
***Conductivity (μScm^−1^)***	12900	10525	6680	6050	24500	16650
***DO (mg/L)***	8.17	3.6	5.03	3.4	10	3.8
***Turbidity (Formazin Turbidity Unit)***	800	690	795	700	3550	2500
***TSS (mg/L)***	1000	620	1400	245	7100	5200
***TDS (mg/L)***	8290	3110	4250	3025	12500	8325
***COD (mg/L)***	125	61	109	62	290	63
***BOD (mg/L)***	60	32	51	33	150	34
***Cadmium (mg/L)***	0.337	0.124	0.183	0.094	0.030	0.014
***Nickel (mg/L)***	0.004	0.002	0.037	0.016	0.012	0.005
***Zinc (mg/L)***	0.139	0.082	0.139	0.111	1.690	0.940

Abbreviations: DO, Dissolved oxygen; TSS, Total suspended solids; TDS, Total dissolved solids

**Figure 1:**
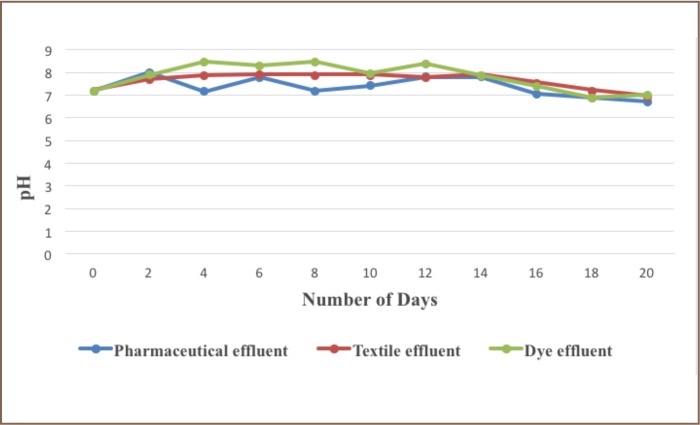
pH of the three samples following biodegradation

#### Fungi

The microorganism used for biodegradation was Penicillium chrysogenum, since it is common to all three effluents. There was a reduction in the pH of the three effluents after 21 days. The pH of the pharmaceutical effluent reduced from 5.6-5.33, the pH of the textile effluent reduced from 5.60-4.92, and the pH of the dye effluent reduced from 5.60-5.26 after 21 days.

**Figure 2:**
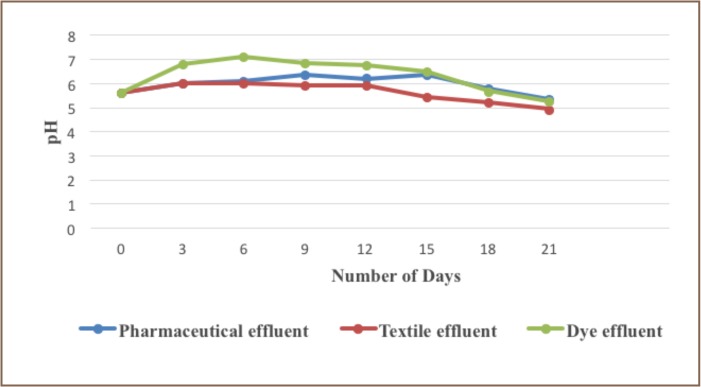
Graph of pH for the three samples after fungi degradation

## Discussion

In this study, the results of the physicochemical analysis of the three effluents showed levels of almost all the analyzed parameters to be higher than WHO permissible limits.^27^,^28^ This is in agreement with a report by Lokhande et al., which stated the physicochemical parameters of effluent was greatly increased in the paint, pharmaceutical and dye industry effluent compared with permissible limits.^30^ Heavy metal analysis in the three samples revealed the presence of cadmium, nickel and zinc, which may be because that these metals form part of the chemical constituents of various mixtures used as by-products in these industries. Similar findings were also reported by Anyakora et al., who stated that the concentration of different metals in the effluent varied significantly, giving credence to the idea that these metals are part of the manufacturing process.^31^ Cadmium was detected in the highest concentration in the sample of pharmaceutical effluent. The concentration of nickel was also revealed to be the highest in the textile effluent, followed by local dye effluent, whereas zinc was observed to be highest in local dye effluent. This was in line with the findings of Ogunleye et al., which reported an increase in the heavy metal concentration in some analyzed industrial effluents, beyond the permissible limits.^32^ In addition, Obasi et al., similarly reported the presence of zinc and nickel in pharmaceutical waste water from Lagos Metropolitan Area.^33^ These metals are known to be readily soluble in water, which makes them available for aquatic life to take up. These metals can also be taken up into the fat tissues of aquatic organisms and become magnified along the food chain, which may lead to detrimental health issues if the there is no proper check on industrial discharges. This was reported by Haman and Bottcher, who stated that long-term exposure to cadmium can cause serious damage to the liver, kidney, bone and blood.^34^

The microbial analysis of the samples revealed the presence of four bacteria: Bacillus cereus, Salmonella typhi, Kleb pneumoniae, Bacillus subtilis and two fungi: Aspergillus flavus, and Penicillium sp. B. subtilis. Aspergillus flavus and Penicillium chrysogenum were isolated in pharmaceutical effluent, Aspergillus flavus, Penicillium chrysogenum, Bacillus cereus, Salmonella typhi and Kleb pneumoniae were observed in the textile effluent, while local dye effluent contained Penicillium chrysogenum, Bacillus subtilis and Bacillus cereus. The pharmaceutical effluent was biodegraded using Bacillus subtilis and Penicillium chrysogenum, and a reduction in value of physicochemical parameters and heavy metals concentration was observed. Biodegradation of the textile effluent by Salmonella typhi and Penicillium chrysogenum showed a reduction in the initially recorded values of physicochemical parameters and heavy metals. This shows that the bacteria and fungi used were capable of breaking down and utilizing these pollutants with low or no impact on the various components of the aquatic environment. These results are in agreement with those of Joutey et al., who reported that microbes that inhabit the soil and groundwater utilize some pollutant chemicals for food and when they completely digest the chemicals and change them into water and harmless gases.^35^ The reduction of the physicochemical values by the isolated microbes may be due to consumption of inorganic and organic matter by microbes for food, a conclusion supported by the work of Elizabeth et al. and Noorjahan and Jamuna.^36^,^37^

## Conclusions

Most industrial effluents contain hazardous chemicals that may have direct or indirect impacts on aquatic biota by bioaccumulation along the food chain and which may later become biomagnified. Many heavy metals that are found in these effluents have been shown by previous studies to be toxic to both aquatic fauna and flora and therefore stricter regulation of these industries is needed.^38^ This present study confirmed the capability of different microbes (Bacillus subtili, Salmonella typhi and Bacillus cereus) to break down the pollutants in three effluents, pharmaceutical, textile and local dye, to a less toxic form. These microbes should be enhanced in their natural ecosystem in other to be able to degrade more of these pollutants. This method should be embraced because of its advantage over other methods; it is environmentally friendly, lower cost, equally effective, and able to bring about a cleaner and more sustainable ecosystem.

## References

[1] Alam A, Sharma V Environmental biotechnology – a review. Researcher [Internet]. 2013 3 27 [cited 2016 Nov 21]; 5 4: 71– 93. Available from: http://www.sciencepub.net/researcher/research0504/013_17507research0504_71_93.pdf

[2] Bhatnagar S, Kumari R Bioremediation: a sustainable tool for environmental management – a review. Annu Rev Res Biol [Internet]. 2013 Oct-Dec [cited 2016 Nov 21]; 3 4: 974– 93. Available from: http://www.journalrepository.org/media/journals/ARRB_9/2013/Aug/1376201429-Bhatnagar342013ARRB5194.pdf

[3] Suteu D, Zaharia C, Bilba D, Muresan R, Popescu A, Muresan A. Decolorization wastewaters from the textile industry-physical methods, chemical methods. Industria Textila [Internet]. 2009 10 [cited 2016 Nov 21]; 60 5: 254– 63. Available from: https://www.researchgate.net/publication/267394522_Decolorization_wastewaters_from_the_textile_industry_-_physical_methods_chemical_methods Romanian.

[4] Babu BR, Parande AK, Raghu S, Kumar TP. Cotton textile processing: waste generation and effluent treatment. J Cotton Sci [Internet]. 2007 1 [cited 2016 Nov 21]; 11 3: 141– 53. Available from: http://www.cotton.org/journal/2007-11/3/upload/jcs11-141.pdf

[5] Henry MP, Donlon BA, Lens PN, Colleran EM. Use of anaerobic hybrid reactors for treatment of synthetic pharmaceutical wastewaters containing organic solvents. J Chem Technol Biotechnol [Internet]. 1996 7 [cited 2016 Nov 21]; 66 3: 251– 64. Available from: http://onlinelibrary.wiley.com/doi/10.1002/(SICI)1097-4660(199607)66:3%3C251::AID-JCTB496%3E3.0.CO;2-S/abstract Subscription required to view.

[6] Huantian C, Ian R Optimization of conditions for microbial decolorization of textile wastewater: starch as carbon source. AATCC Rev [Internet]. 2001 10 [cited 2016 Nov 21]; 1 10: 37– 42. Available from: http://connection.ebscohost.com/c/articles/31857651/optimization-conditions-microbial-decolorization-textile-wastewater-starch-as-carbon-source Subscription required to view.

[7] Vilaseca M, Gutierrez MC, Lopez-Grimau V, Lopez-Mesas M, Crespi M. Biological treatment of a textile effluent after electrochemical oxidation of reactive dyes. Water Environ Res [Internet]. 2010 2 [cited 2016 Nov 21]; 82 2: 176– 82. Available from: http://www.ingentaconnect.com/content/wef/wer/2010/00000082/00000002/art00009 Subscription required to view. 10.2175/106143009x447902 20183984

[8] Awomeso AJ, Taiwo AM, Gbadebo AM, Adenowo JA. Studies on the pollution of waterbody by textile industry effluents in Lagos, Nigeria. J Appl Sci Environ Sanitation [Internet]. 2010 Oct-Dec [cited 2016 Nov 21]; 5 4: 353– 9. Available from: https://www.researchgate.net/publication/49604247_Studies_on_the_pollution_of_waterbody_by_textile_effluents_inLagos_Nigeria

[9] Tufekci N, Sivri N, Toroz I. Pollutants of textile industry wastewater and assessment of its discharge limits by water quality standards. Turkish J Fish Aquat Sci [Internet]. 2007 [cited 2016 Nov 21]; 7: 97– 103. Available from: http://www.trjfas.org/uploads/pdf_319.pdf

[10] Yusuff RO, Sonibare JA Characterization of textile industries effluents in Kaduna, Nigeria and pollution implications. Global Nest J [Internet]. 2004 [cited 2016 Nov 21]; 6 3: 212– 21. Available from: http://www.academia.edu/912996/Characterization_of_textile_industries_effluents_in_Kaduna_Nigeria_and_pollution_implications

[11] Brown D, Laboureur P The aerobic biodegrability of primary aromatic amines. Chemosphere [Internet]. 1983 [cited 2016 Nov 21]; 12 3: 405– 14. Available from: http://www.sciencedirect.com/science/article/pii/0045653583901157 Subscription required to view.

[12] Das N, Chandran P Microbial degradation of petroleum hydrocarbon contaminants: an overview. Biotechnol Res Int [Internet]. 2011 [cited 2016 Nov 21]; 2010: 1– 13. Available from: https://www.hindawi.com/journals/btri/2011/941810/ 10.4061/2011/941810 PMC3042690 21350672

[13] Pramila R, Padmavathy K, Ramesh KV, Mahalakshmi K. Brevibacillus parabrevis, Acinetobacter baumannii and Pseudomonas citronellolis - potential candidates for biodegradation of low density polyethylene (LDPE). J Bacteriol Res [Internet]. 2012 3 [cited 2016 Nov 21]; 4 1: 9– 14. Available from: http://www.academicjournals.org/article/article1380020448_Pramila%20et%20al.pdf

[14] El Fantroussi S, Agathos SN Is bioaugmentation a feasible strategy for pollutant removal and site remediation? Curr Opin Microbiol [Internet]. 2005 6 [cited 2016 Nov 21]; 8 3: 268– 75. Available from: http://www.sciencedirect.com/science/article/pii/S1369527405000512 Subscription required to view. 10.1016/j.mib.2005.04.011 15939349

[15] Zapata A, Oller I, Sirtori C, Rodriguez A, Sanchez-Perez JA, Lopez A, Mezcua M, Malato S. Decontamination of industrial wastewater containing pesticides by combining large-scale homogeneous solar photocatalysis and biological treatment. Chem Eng J [Internet]. 2010 6 1 [cited 2016 Nov 21]; 160 2: 447– 56. Available from: http://www.sciencedirect.com/science/article/pii/S1385894710002615 Subscription required to view.

[16] Kavitha V, Palanivelu K The role of ferrous ion in Fenton and photo-Fenton processes for the degradation of phenol. Chemosphere [Internet]. 2004 6 [cited 2016 Nov 21]; 55 9: 1235– 43. Available from: http://www.sciencedirect.com/science/article/pii/S0045653504000487 Subscription required to view. 10.1016/j.chemosphere.2003.12.022 15081764

[17] Bertea A, Bertea AP Decolorisation and recycling of textile wastewater. Iasi, Romania: Performantica Publishing House; 2008 Romanian.

[18] Bisschops I, Spanjers H Literature review on textile wastewater characterisation. Environ Technol [Internet]. 2003 11 [cited 2016 Nov 21]; 24 11: 1399– 411. Available from: http://www.tandfonline.com/doi/abs/10.1080/09593330309385684 Subscription required to view. 10.1080/09593330309385684 14733393

[19] Standard Methods for the Examination of Water and Wastewater ( : Clesceri LS, Greenberg AE Eaton AD), 20th Edition, ISBN: 0875532357 American Public Health Association 1999 Washington, D.C., 1325 p.

[20] Correia VM, Stephenson T, Judd SJ. Characterization of textile wastewater – a review. Environ Technol [Internet]. 1994 7 11 [cited 2016 Nov 21]; 15: 10: 917– 29. Available from: http://www.tandfonline.com/doi/abs/10.1080/09593339409385500 Subscription required to view.

[21] Orhon D, Babuna FG, Insel G. Characterization and modelling of denim-processing wastewater for activated sludge. J Chem Technol Biotechnol [Internet]. 2001 9 [cited 2016 Nov 21]; 76 9: 919– 31. Available from: http://onlinelibrary.wiley.com/wol1/doi/10.1002/jctb.462/full Subscription required to view.

[22] Clescerl LS, Greenberg AE, Eaton AD, Standard methods for examination of water and wastewater. 20th ed. New York: American Public Health Association; 1999 Jan. 1325 p.

[23] Chikere CB, Okpokwasili GC, Chikere BO. Bacterial diversity in a tropical crude oil-polluted soil undergoing bioremediation. Afr J Biotechnol [Internet]. 2009 6 3 [cited 2016 Nov 21]; 8 11: 2535– 40. Available from: http://www.ajol.info/index.php/ajb/article/download/60762/48983

[24] Barnett HL, Hunter BB Illustrated genera of imperfect fungi. 3rd Ed. Minneapolis, MN: Burgress Publication Company; 1972 241 p.

[25] Orji FA, Ibiene AA, Okerentugba PO. Bioremediation of petroleum hydrocarbon-polluted mangrove swamps using nutrient formula produced from water hyacint (Eicchornia crassipes). Am J Environ Sci [Internet]. 2013 [cited 2016 Nov 21]; 9 4: 348– 66. Available from: http://thescipub.com/PDF/ajessp.2013.348.366.pdf

[26] Chukwura EI, Nwokolo CI, Nwachukwu SC. Bioremediation of crude oil polluted Escravos River using Candida utilis. Nigerian J Microbiol. 2005; 19: 623– 30

[27] International Standards for Drinking Water, 3rd ed. 1986 World Health Organization, Geneva, Switzerland

[28] Guidelines for Drinking Water Quality. ( 4th Edition). Volume 1, 2011 World Health Organization, Geneva, Switzerland http://apps.who.int/iris/bitstream/10665/44584/1/9789241548151_eng.pdf

[29] Nigerian standards for drinking water quality (NSDWQ), Industrial standards 554, 1-14, 2007. Unicef/Standards Organization of Nigeria, Lagos, Nigeria https://www.unicef.org/nigeria/ng_publications_Nigerian_Standard_for_Drinking_Water_Quality.pdf

[30] Lokhande RS, Singare PU, Pimple DS. Study on physico-chemical parameters of waste water effluents from Taloja Industrial Area of Mumbai, India. Int J Ecosyst [Internet]. 2011 [cited 2016 Nov 21]; 1 1: 1– 9. Available from: http://article.sapub.org/10.5923.j.ije.20110101.01.html

[31] Anyakora C, Nwaeze K, Awodele O, Nwadike C, Arbabi M, Coker H. Concentrations of heavy metals in some pharmaceutical effluents in Lagos, Nigeria. J Environ Chem Ecotoxicol [Internet]. 2011 2 [cited 2016 Nov 21]; 3 2: 25– 31. Available from: http://www.academicjournals.org/journal/JECE/article-full-text-pdf/5F08E961339

[32] Ogunleye IO, Izuagie AA Determination of heavy metal contents in some industrial effluents from Ondo State, Nigeria. J Environ Chem Ecotoxicol [Internet]. 2013 8 [cited 2016 Nov 21]; 5 8: 216– 9. Available from: http://www.academicjournals.org/journal/JECE/article-full-text-pdf/B2929E74274

[33] Obasi AI, Amaeze NH, Osoko DD. Microbiological and toxicological assessment of pharmaceutical wastewater from the Lagos Megacity, Nigeria. Chin J Biol [Internet]. 2014 [cited 2016 Nov 21]; 2014: 1– 9. Available from: https://www.hindawi.com/archive/2014/638142/

[34] Haman DZ, Bottcher DB Home water quality and safety [Internet]. Gainesville, FL: University of Florida; 1986 5 [cited 2016 Nov 21] 15 p. Available from: http://www.pinecrest-fl.gov/Modules/ShowDocument.aspx?documentid=2809

[35] Joutey NT, Bahafid W, Sayel H, El Ghachtouli N. Biodegradation: involved microorganisms and genetically engineered microorganisms. : Chamy R, Rosenkranz F, Biodegradation - life of science [Internet]. Rijeka, Croatia: InTech; 2013 6 14 [cited 2016 Nov 21]. Chapter 11. Available from: http://www.intechopen.com/books/biodegradation-life-of-science/biodegradation-involved-microorganisms-and-genetically-engineered-microorganisms

[36] Elizabeth KM, Ambica T, Vimala Y. Physicochemical and microbiological parameters of beer manufacturing industrial effluent: bioremediation of pollutants. Poll Res. 2006; 25 2: 273– 6.

[37] Noorjahan CM, Jamuna S Physico-chemical characterisation of brewery effluent and its degradation using native fungus - aspergillus niger, aquatic plant - water hyacinth -eichhornia SP and green mussel - pernaviridis. J Environ Earth Sci [Internet]. 2012 5 [cited 2016 Nov 21]; 2 4: 31– 40 Available from: https://www.researchgate.net/publication/225292636_Physico-Chemical_Characterisation_of_Brewery_Effluent_and_Its_Degradation_using_Native_Fungus_-Aspergillus_Niger_Aquatic_Plant_-Water_Hyacinth-Eichhornia_SP_and_Green_Mussel_-_Pernaviridis

[38] Stalikas CD, AChMantalovas, Pilidis G.A. Multi element concentrations in vegetable species grown in two typical agricultural areas of Greece. Science of the Total Environment. 1997; 206: 17– 24 9373989 10.1016/s0048-9697(97)00213-1

